# Morphology of Influenza B/Lee/40 Determined by Cryo-Electron Microscopy

**DOI:** 10.1371/journal.pone.0088288

**Published:** 2014-02-06

**Authors:** Garrett Katz, Younes Benkarroum, Hui Wei, William J. Rice, Doris Bucher, Alexandra Alimova, Al Katz, Joanna Klukowska, Gabor T. Herman, Paul Gottlieb

**Affiliations:** 1 Department of Microbiology and Immunology, Sophie Davis School of Biomedical Education, The City College of New York, New York, New York, United States of America; 2 Department of Computer Science, The Graduate Center, City University of New York, New York, New York, United States of America; 3 The New York Structural Biology Center, New York, New York, United States of America; 4 Department of Microbiology and Immunology, New York Medical College, Valhalla, New York, United States of America; 5 Department of Physics, The City College of New York, New York, New York, United States of America; University of Edinburgh, United Kingdom

## Abstract

Cryo-electron microscopy projection image analysis and tomography is used to describe the overall architecture of influenza B/Lee/40. Algebraic reconstruction techniques with utilization of volume elements (blobs) are employed to reconstruct tomograms of this pleomorphic virus and distinguish viral surface spikes. The purpose of this research is to examine the architecture of influenza type B virions by cryo-electron tomography and projection image analysis. The aims are to explore the degree of ribonucleoprotein disorder in irregular shaped virions; and to quantify the number and distribution of glycoprotein surface spikes (hemagglutinin and neuraminidase) on influenza B. Projection image analysis of virion morphology shows that the majority (∼83%) of virions are spherical with an average diameter of 134±19 nm. The aspherical virions are larger (average diameter = 155±47 nm), exhibit disruption of the ribonucleoproteins, and show a partial loss of surface protein spikes. A count of glycoprotein spikes indicates that a typical 130 nm diameter type B virion contains ∼460 surface spikes. Configuration of the ribonucleoproteins and surface glycoprotein spikes are visualized in tomogram reconstructions and EM densities visualize extensions of the spikes into the matrix. The importance of the viral matrix in organization of virus structure through interaction with the ribonucleoproteins and the anchoring of the glycoprotein spikes to the matrix is demonstrated.

## Introduction

The influenza viruses are members of the *orthomyxoviridae* family of which there are three distinct types - A, B, and C. Both types A and B produce epidemic disease and type A produces pandemic disease. In some years, B has higher prevalence than A [1], [2]. Therefore both A (two subtypes) and type B are included in the trivalent seasonal influenza vaccine. Since two distinct antigenic lineages of B exist with varying incidence, manufacturers have gained approval to formulate quadrivalent vaccines [2]. Due to the antigenic diversity that is seen in influenza viruses, new vaccines must be reformulated on an annual schedule. Influenza A and B both contain segmented genomes composed of 8 distinct ribonucleoprotein (RNP) elements, each containing a negative sense RNA [3]. The surface glycoprotein antigens, hemagglutinin (HA) and neuraminidase (NA) are both significant immunogens that contribute to the development of an anti-influenza response [4]. In addition to understanding basic virus structure and properties of these viruses, it would also be of value to vaccine manufactures to determine the quantity and relative amounts of HA and NA on the virion surface of wild type and vaccine candidates. This would aid in determining the best candidate for maximal antigen yield during vaccine production. Furthermore, the quantities and relative amounts of the HA and NA will affect efficacy of the vaccine in producing an immunogenic response. The virus particle shape may also be of importance for high yield production as strains with spherical virions are known to produce higher titer *in ovo* than strains which are filamentous [5]. Spherical virions may have the biophysical properties necessary for optimal purification of the virus during the gradient centrifugal steps utilized during preparation of the vaccine. Ruigrok et al. [6] found that spherical influenza particles had higher infectivity and a higher ratio of HA to M protein than non-spherical virions. High HA spike quantity is an important property for efficient vaccine production.

X-ray crystallography has revealed the atomic structure of HA [7]–[10] and the NA head [11], [12]. HA are trimers with an elongated fusion domain, a globular receptor-binding domain and a vestigial esterase domain. NA are club-shaped tetramers with 4-stranded anti-parallel β-sheets arranged like the blades of a propeller [11], [12].

Influenza particles are pleomorphic. Laboratory adapted strains are typically spherical with diameters in the range of 100 to 130 nm, but the virions can also exist as larger ellipsoids or filamentous virions which can be several microns in length [13], [14]. Because influenza is pleomorphic, 3-D structural analysis of the entire virion is not possible by single particle analysis or tomogram averaging. Cryo-EM tomography has been employed to study the surface proteins. Harris *et al* [15] visualized the 3-D structure of type A H3N2 strain X-31 virus using cryo-EM tomography and determined that a typical 120 nm diameter type A influenza virion can contain up to 375 surface spikes but the actual count could be lower due to viral surface regions with lower spike density. Calder et al. [14] employed cryo-EM tomography to study the structural organization of filamentous influenza A and observed that the interaction between the M1 protein and surrounding envelope determines morphology of the virion. Giocondi et al. [16] used atomic force microscopy to study the 3-D topography of H1N1 influenza and a lateral heterogeneity of the HA and NA spikes was observed for virions at neutral pH and after treatment at pH 5. The distributions of surface glycoproteins on two filamentous type A virus particles (A/Udorn/72 and A/Aichi/68 X-31) has recently been determined [17].

Improvements in the reconstruction algorithms may yield better identification of components of this non-symmetric virus. Marabini et al. [18], [19] demonstrated that Algebraic Reconstruction Techniques (ART) using generalized Kaiser-Bessel window functions (commonly known as blobs) produced more efficacious 3-D reconstructions in electron microscopy than those produced by alternative methods. ART with blobs has been shown to be superior to weighted backprojection and computationally more efficient than simultaneous iterative reconstruction techniques (SIRT) [18]–[20]. ART are iterative procedures for solving systems of linear equations or inequalities that were first proposed by Kaczmarz [21] and introduced to the biomedical field by Gordon et al. [22]. Blobs are spherically symmetric volume elements that are superior to voxels for the estimation of the shapes of biological objects and allow for the fact that biological elements usually lack perpendicular edges [18]. Blobs also account for overlap creating smooth transitions, a property useful for reconstruction of influenza virions with the large number of surface spikes. In this work, it is shown that ART offers reasonable resolution and fidelity for the differentiation of the HA and NA surface spikes.

In the work presented, the structure of Influenza B/Lee/40 is investigated by cryo-EM tomography and projection image analysis. B/Lee/40 was used as an influenza vaccine component in the 1940’s and is currently used as the high yield gene donor for preparation of reassortant vaccine candidates with improved growth characteristics *in ovo* [23].

As a first step in the detailed morphological classification of type B/Lee/40 influenza virus, both projection images and tomogram slices reconstructed by ART with blobs of the viral particles were collected and analyzed. Together, they were utilized to characterize the pleomorphic forms of B/Lee/40; determine the relationships of the RNPs to viral shape and size; and differentiate the surface spike protein elements. Our overall goal in this paper is to identify the fundamental structural and morphological features of a type B virus.

It is noted that two types of surface spikes which are resolved in the collected images correspond to the HA trimers and NA tetramers. Additionally the observations are significant in regard to the quantization of the HA content on the viral surfaces of both types A and B influenza viruses.

## Materials and Methods

### Virus Propagation and Purification

B/Lee/40 influenza virus was grown and amplified in embryonated chicken eggs. The original ‘seed’ allantoic fluids, containing type B virions, were diluted 1∶1000 in PBS containing 250 µg/ml gentamicin. Each egg was inoculated with 0.1 ml of the diluted allantoic fluid and incubated at 33°C for 72 h. A step gradient was utilized to purify the virus particles to approximately 1.6 mg/ml protein total viral mass [24]. The eggs were utilized at the New York Medical College. All the work involved embryonated eggs 12 days and under and as such does not require IACUC approval. Embryos were euthanized by hypothermia.

### Cryo-electron Microscopy

#### Grid sample preparation

Three µl of a suspension of Influenza B/Lee/40 sample was placed onto glow-discharged, perforated Quantifoil grids, blotted, and plunge-frozen in liquid ethane. For some experiments, the virus sample was pre-mixed with a suspension of 10 nm gold beads in order to add fiducial markers to aid in tomographic reconstruction.

#### Tomographic image collection

Two electron microscopes were used for this study. Preliminary images were recorded at 25000× magnification and a defocus of 8±0.5 µm, using a Tecnai F20 electron microscope (FEI, Inc, Hillsboro, OR) operating at 200 kV on a TVIPS F415 4096×4096 CCD (Tietz Video and Image Processing Systems GmbH Gauting Germany), binning two-fold (effective pixel size of 8.8 Å/pixel) under low-dose conditions. Subsequent images were recorded at 25000× or 50000× magnification (4.4 and 2.2 Å/pixels prior to binning, respectively) and an underfocus of 8±0.5 µm, with a JEOL 3200FSC electron microscope (JEOL, West Chester, PA) operating at 300 kV which provided improved resolution. An energy filter, with a slit width of 20 eV, was inserted to eliminate non-elastically scattered electrons and thereby enhance contrast. Tilt series were recorded using the SerialEM software [25] on a 4096×4096 pixel CCD camera (Gatan Inc, Pleasanton, CA). Specimen angles ranged between ±60° with 2° steps. The low-dose imaging mode limited total specimen dosage to 60 e/Å^2^ over the entire tilt series. A total of 10 tilt series were acquired containing ∼ 150 virions. Three dimensional reconstructions were performed on 3 isolated virions which were approximately spherical.

#### Tilt-series image processing

For tilt series with fiducial markers, tomographic alignment and reconstruction was performed using IMOD software [26]. Tilt series without fiducial markers were aligned and reconstruction using Protomo software [27]. In this method, alignment of the original projection images is iterated until relative image shifts are less than 1 pixel and geometrical correction factors are within 1%. Both packages use R-weighted backprojection for tilt series alignment in the reconstruction. The best results were achieved using the fiducial markers, and these reconstructions were used to manually identify virion centers in 3D. The center coordinates were reprojected into the tilt series, and then used to excise smaller boxed tilt series for individual particles. After reconstruction, contrast was enhanced by applying a non-linear anisotropic diffusion filter [28] implemented in the SPIDER [29] image processing suite. In general, 60 cycles of this filter was found to yield good results. The boxed tilt series were also used as input for the ART reconstruction in XMIPP [30]. Three dimensional visualizations were generated using Amira software (Visage Imaging, San Diego, CA).

### Algebraic Reconstruction Techniques

A detailed discussion of ART is given in Chapter 11 of the book by Herman [31]. Briefly the idea is the following: the virion to be reconstructed is represented by a function (*f*(*x*, *y*, *z*)) which is described by a linear combination of a finite set of fixed basis functions *b_j_*, such that 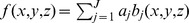. The task is to estimate the unknown coefficients *a_j_* based on projection images. All the pixels in all the projection images are indexed by the integer *i* (1≤ *i* ≤ *I*, where *I* is the total number of pixels in the projection images). Let *p_i_* be the value of the *i* ^th^ pixel; it is approximately the integral of *f*(*x,y,z*) along a line that projects onto the center of the *i* ^th^ pixel. Since the basis function *b_j_*(*x*, *y*, *z*) is known (for 1≤ *j* ≤ *J*), it is possible to calculate the line integral *r_i,j_* of the *j* ^th^ basis function along the *i* ^th^ line. This results in the system of approximate equations
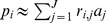
, for 1≤ *i* ≤ *I*. The version of ART used iteratively estimates the unknowns, *a_j_* as follows. The initial value, *a* ^0^ *_j_* is typically assigned the value zero, for 1≤ *j* ≤ *J*. Suppose that the values *a^k^_j_* of the *k* ^th^ iterate are known. To obtain the next iterate, an *i* is picked (typically by repeatedly cycling through all the *i’*s in some order) and defined by:
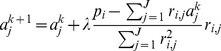
(1)where λ is a positive number (a typical value for electron tomography would be 0.05). That this method is efficacious for the tomographic reconstructions of virions when blobs are used as the *b_j_* can be seen by observing the quality of the images shown in the Results Section.

### Polar Plot Correlations

Polar plots of central tomogram slices were converted to Cartesian coordinates using Chimera software [32]. Since the virions are not precise spheres, first, step-wise linear shifts were applied in the radial (y-) direction to align the outer membrane at a near constant radius. Next, segments, ten rows thick (∼1.67 nm), were averaged to reduce noise. The correlation coefficient *C_jk_* between the *r_j_* and *r_k_* segments is given by:
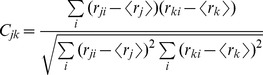
(2)where *i* is the column (x-axis) index were calculated between the segment corresponding to the heads of the protein spikes; and all other segments at radii greater than the inner boundary of the membrane.

## Results and Discussion

### Morphology and Size Distribution

**Figure 1:**
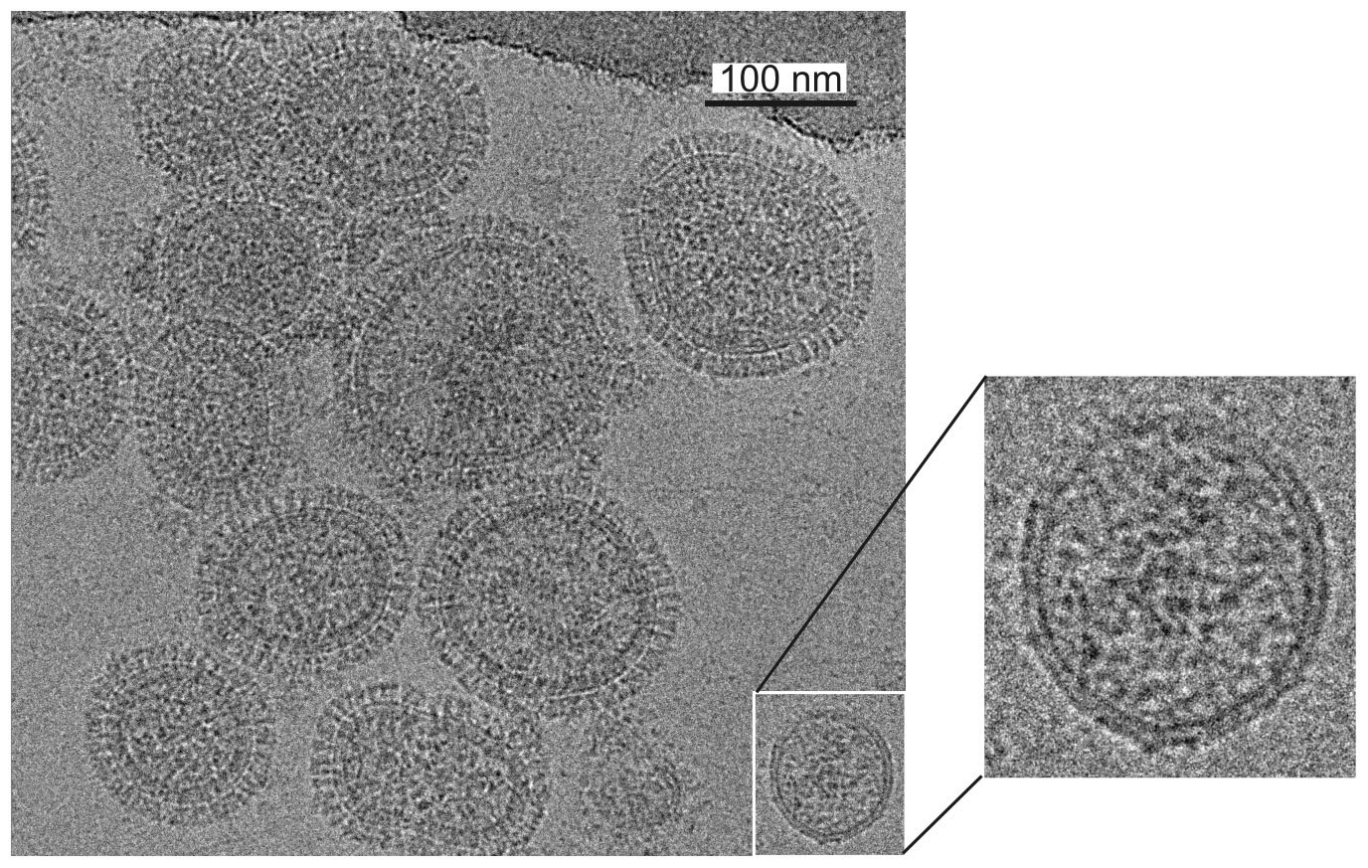
Projection image of frozen-hydrated influenza B virions. Surface spike are visible in all particles with intact or partially intact matrix. A defective virion which lacks surface proteins is identified in lower right and enlarged in inset.

**Figure 2:**
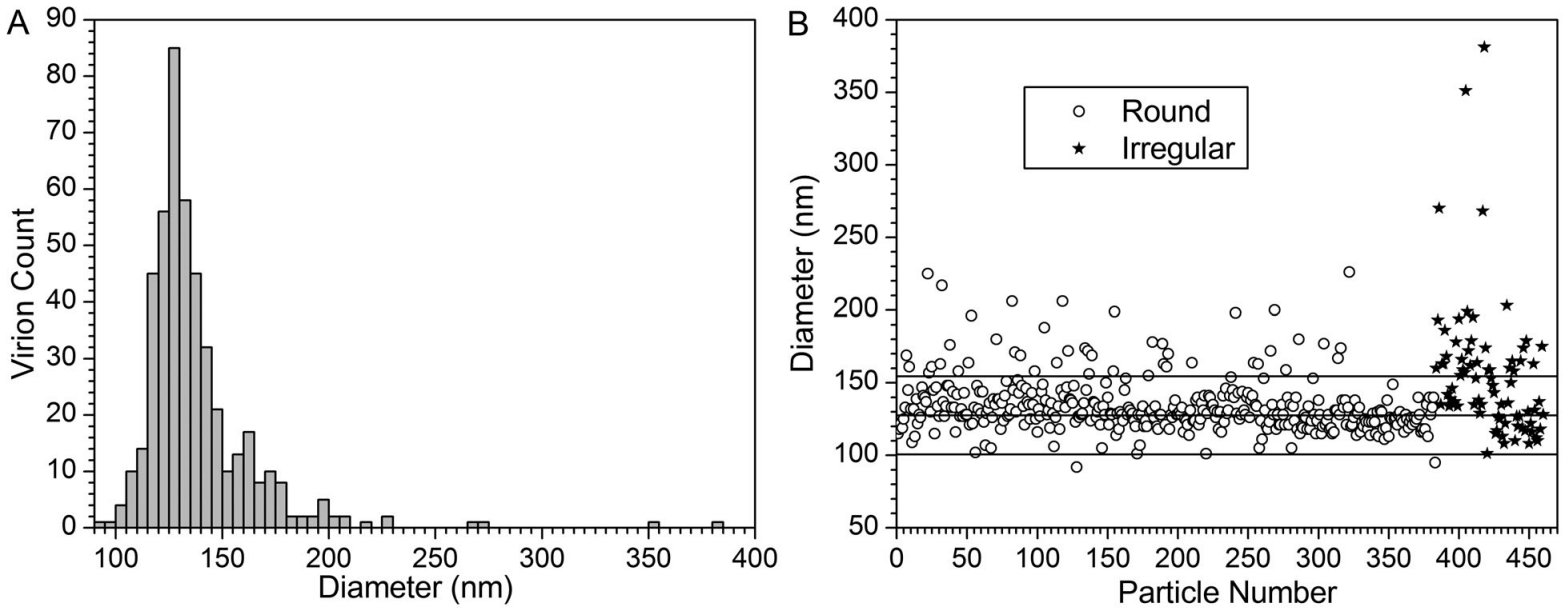
Virion Size Distribution. A) Histogram of virion diameter. B) Scatter plot of virion diameter; horizontal lines are mean and mean ±1 standard deviation.

The examination of conventional projection images of frozen-hydrated Influenza B/Lee/40 viruses show intact particles, each surrounded by a distinct envelope that contains the surface glycoprotein spikes, HA and NA. The particles in these initial projection images are not uniformly sized and several had an oblong configuration as can be seen in Fig. 1. In the majority of the virions, the periphery is well defined and consists of the outer lipid bilayer with the matrix underneath. In addition to the entirely assembled viruses, incompletely assembled particles are evident. The incompletely assembled virions often lack the surface proteins and consist primarily of the lipid envelope (see insert in Fig. 1). A subset of projection images containing 460 intact virions were selected for size analysis. Virion size measurements were performed using projection images because virion diameters can be determined as accurately from projection image analysis as from tomograms and a much larger sample size can be efficiently analyzed. Virions lacking surface proteins or appearing defective were excluded from the size analysis. It was determined that the average virion diameter is 137±27 nm. The size distribution is shown in Fig. 2(A). Of the intact virions, 83% (383 of 460 virions) appear approximately circular in projection images – indicative of spherical particles. The other 17% of the intact virions (77 of 460 virions) are irregular in shape. The irregular virions average 16% larger in size (155±47 nm) than the spherical virions (134±19 nm). The irregular virions exhibit a greater size variation (see Fig. 2(B)) than spherical virions with 10 of 77 irregular-shaped virions (13%) being more than 2 standard deviations larger than the mean, compared to 10 of 383 (3%) of spherical virions. For irregular shaped virions, the size is taken to be the longest linear dimension including the surface glycoproteins; for approximately spherical virions, size is virion diameter including surface glycoproteins.

**Figure 3:**
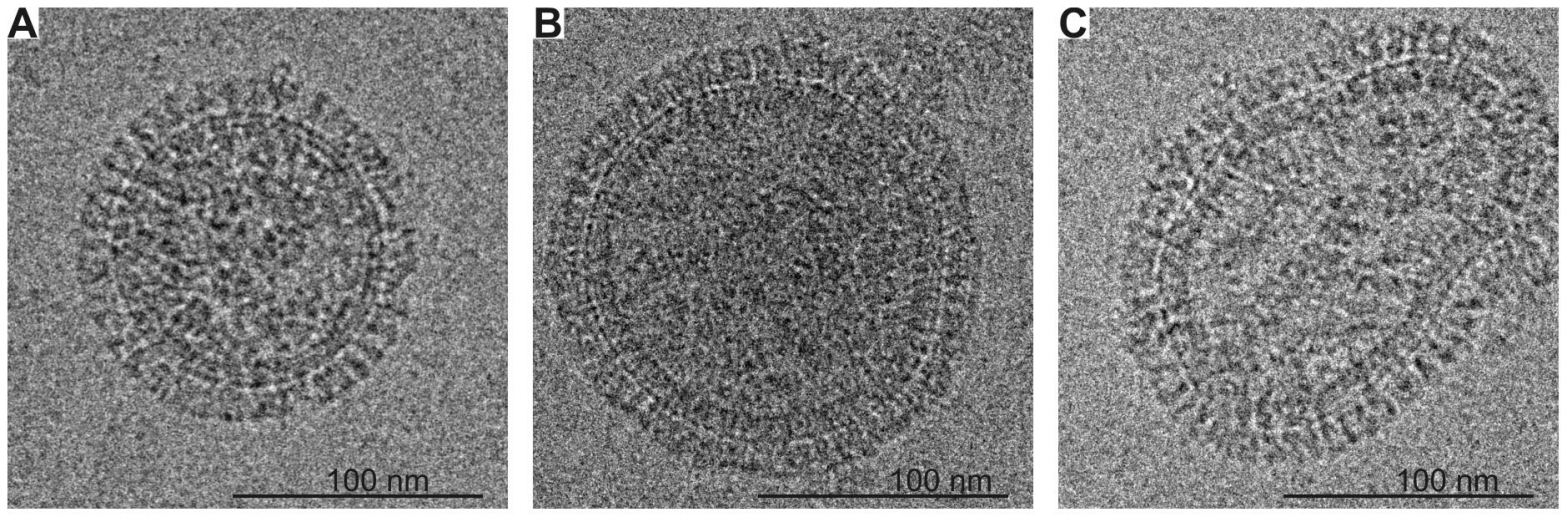
Projection images of three virions. A) Average-size spherical virion; B) large spherical virion; and C) irregular virion. The irregular virion exhibits considerable disruption in the RNPs indicated by low density regions.

To emphasize the morphological variation, selected individual virions are shown in Figs. 3A–C with an average-size spherical in Fig. 3A, a larger-size spherical in Fig. 3B and an intact irregular-shape virion in Fig. 3C. At 50000× magnification, the glycoprotein spikes are clearly resolved. The spikes extend 14.5±1.3 nm beyond the virion membrane, in agreement with reported measurements for type A influenza [33]. Within the completely assembled particles, the RNPs appear with a granular consistency in the projection images but resolved to individual segments in tomogram reconstructions (see cryo-EM tomography section). The irregular virion projection (Fig. 3C) shows several low density regions which may be lacking RNPs densities while the regular-shaped virions show a consistency of internal density. The differences in RNP density distribution between the regular- and irregular-shaped virions may point to the existence of an association between the membrane and the RNP structure.

### Estimate of Glycoprotein Spacing from Projection Images

Approximately 40 spikes are visible in the 100 nm diameter (130 nm including the surface spikes) virion shown in Fig. 3A. If the effects of overlap are excluded from the analysis, the average spacing between protein spikes is 7.9 nm. Assuming each spike occupies an area of (7.9)^2^ nm^2^, approximately 500 total spikes fit on a 130 nm virion [surface area of the envelope is 4π((130–29)/2)^2^ = 3.2×10^4^ nm^2^]. This estimate is slightly higher than that reported for type A influenza by Harris et al. [15] (∼375 glycoprotein spikes on a 120 nm virion), but agrees more closely with the 530 spikes reported for type B/Hong Kong by Booy et al. [34]. Booy’s calculations were also from projection images and the issue of overlap was ignored. The surface spike count on a central tomogram slice of an equal-sized B/Lee/40 virion is 38, implying an average spike spacing of ∼8 nm and, therefore a total of approximately 400 spikes; indicating that the spike count error due to overlap in projection images is about 10% and a typical 130 nm diameter virion contains ∼ 460 surface glycoproteins. However, the poorer z-axis resolution of tomography may lead to an additional underestimation of the overlap.

### Cryo-EM Tomography Reveals Structural Elements of the Virion

**Figure 4:**
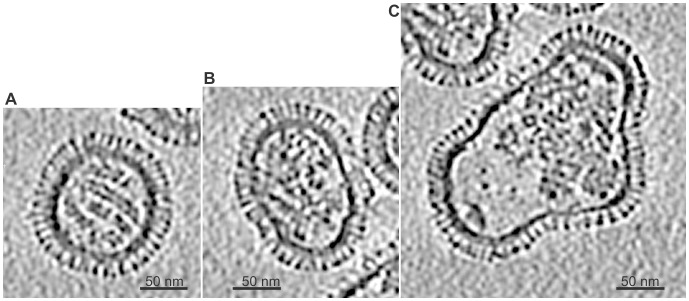
Morphological classes of B/Lee/40 virions. Central tomogram slices created with IMOD using R-weighted backprojections: A) spherical virion with well-ordered RNPs; B) oblong virion with some RNP disorder; and C) Irregular-shaped virion with little RNP order and a large region showing a very low density of RNP (lower left of virion).

Cryo-electron tomography was used to visualize the relationships among the virion elements. In the 7-Å-thick sections, discrete RNP segments are discernible; the viral matrix is well delineated; and the spike projections protruding from the membrane surface are suggestive of the tetrameric (NA) and trimeric (HA) elements. The IMOD R-weighted backprojection tomograms reveal three unique morphological configurations as can be observed in the central slices shown in Figs. 4(A–C). The three configurations are consistent with the projection image observations. The virion in Fig. 4A, with near spherical morphology, shows a distinct organization of RNPs, typical of the reconstructed tomograms of spherical particles. The near spherical configuration is the most abundant with the RNP segments occupying almost the entire volume of the particle’s interior. The second configuration consisted of oblong virions (Fig. 4B). These contained a well-defined envelope-matrix layer entirely covered by the spikes however the RNPs exhibit some disorder. The third configuration consists of larger and highly irregular-shaped virions (Fig. 4C). The RNP segments showed little order with large vacant areas. This is characteristic of the irregular virions.

**Figure 5:**
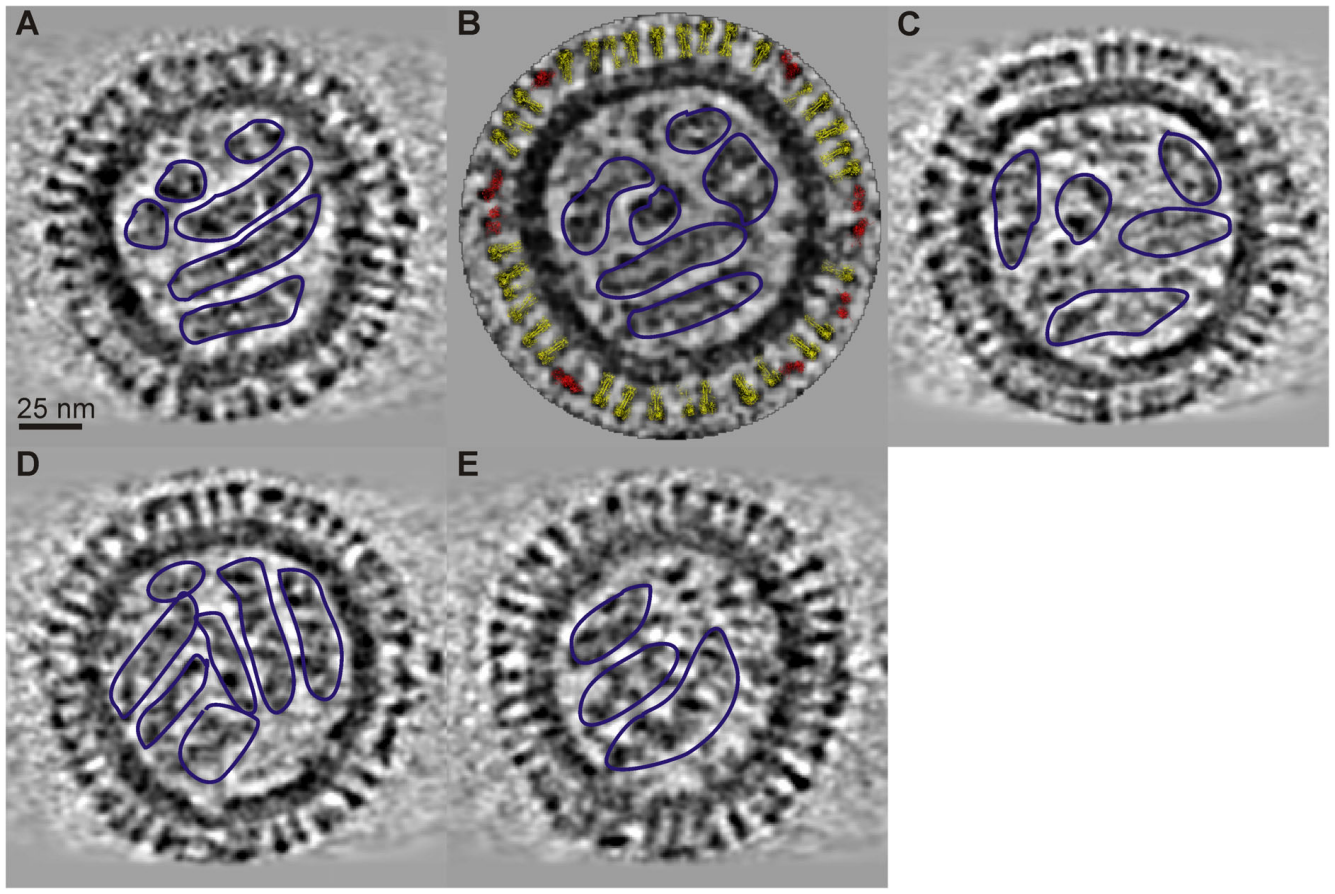
ART Reconstruction of B/Lee/40. A–E) Tomogram slices at 9 nm z-axis spacing. Docked atomic models of HA (yellow) and NA (red) are shown in panel B. RNPs are outlined in blue.

**Figure 6:**
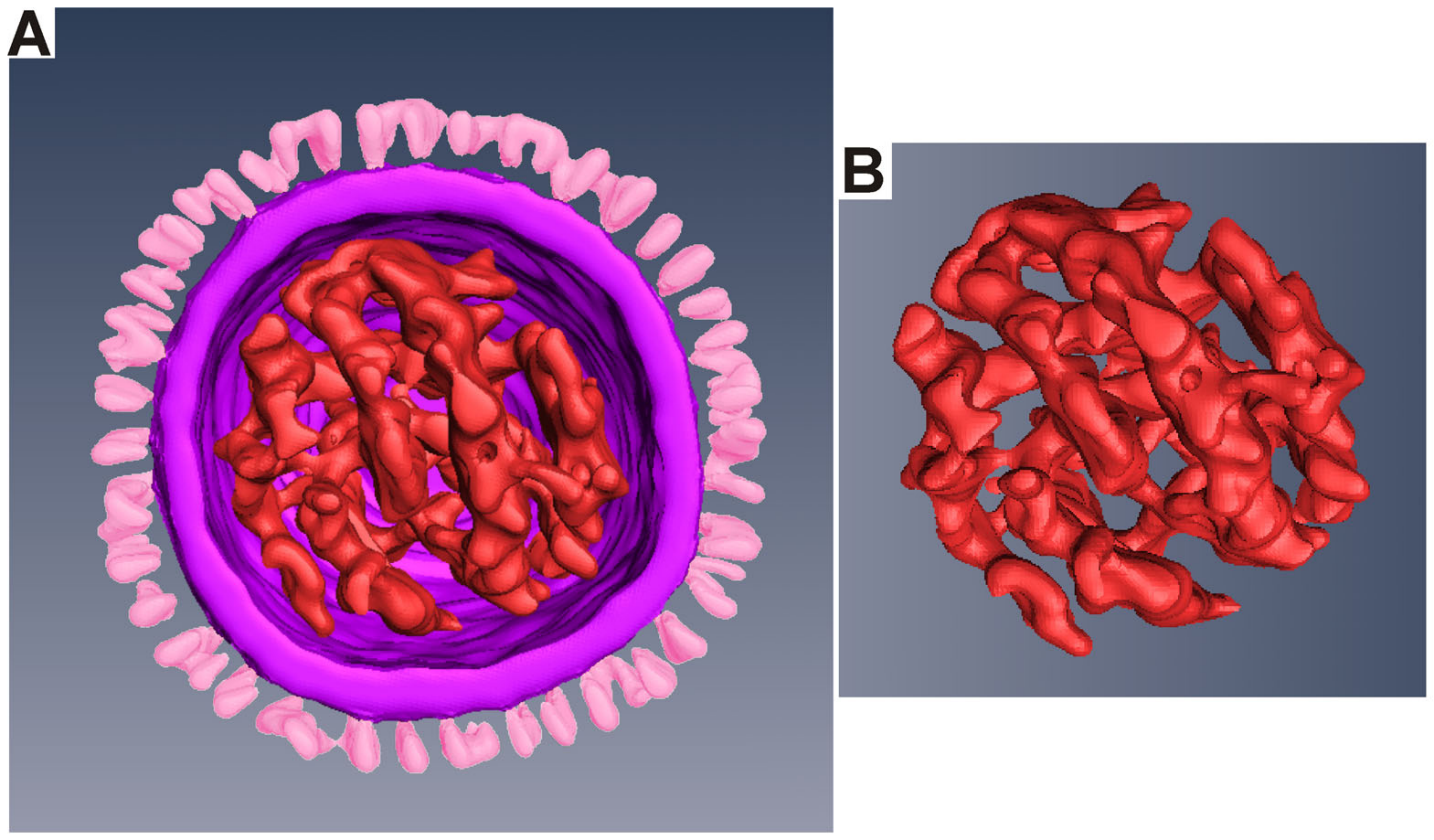
Amira Rendering of the RNPs by segment selection. A) Complete virion. B) RNP elements exclusively presented showing the twisted confirmation.

Midsection tomogram ART slices are shown in Figs. 5A–E for a 125 nm diameter virion. The five slices have a vertical (z-axis) separation of 9 nm. The slices clearly show 14 nm-long distinct surface spikes, an 8-nm thick envelope-matrix, and discrete RNPs inside the virion. The surface proteins penetration into the matrix is resolved and close visual inspection reveals two distinct surface protein morphologies: (1) near uniform density and thickness; and (2) “club-like” and carrying a denser top. The bi-lobed HA trimer is distinguished from the club-like NA tetramer with reasonable accuracy by evaluation of the contour spike density in the tomogram slice. A ribbon rendering of 3-D atomic models of HA (yellow) and NA (red) (PDB entries 2RFT and 1BJI, respectively) [35], [36] are docked into the protein surface spikes using Chimera and shown in Fig. 5B. The correspondence of the docked ribbon to the EM density confirms the identification of these spike types. The central slice in Fig. 5B contains 9 NA and 33 HA indicating 120 NA and 441 HA for a total of 561 glycoprotein spikes on the surface. The orientation of the RNPs is evident in the tomogram slices. The RNPs appear to make contact with the inner matrix and may interact with it. Since the larger, irregular-shaped virions have internal vacant areas, it suggests that breaking the contact between the inner matrix and the RNPs disrupts the overall RNP configuration. Harris et al. [15] reports that in type A influenza the RNP segments are also in contact with the inner matrix at discrete points. It is conceivable that the expanded matrix of atypical-large virion disrupts an RNP attachment site, leading to increased RNP disorder. The type B tomograms do not show the 7 plus 1 configuration for RNP segments as reported for type A during budding [15], [37]. The large irregular particles show some clustering of the RNP in spite of the overall disorder, suggesting the RNP segments may have some attachment to each other. Fig. 6A shows an Amira rendering of the virion (slices shown in Fig. 5) with the isolated RNPs shown in Fig. 6B. The RNP orientations vary across the different the tomogram slices (Fig. 5), implying a twisted conformation which is observed in Fig. 6B.

**Figure 7:**
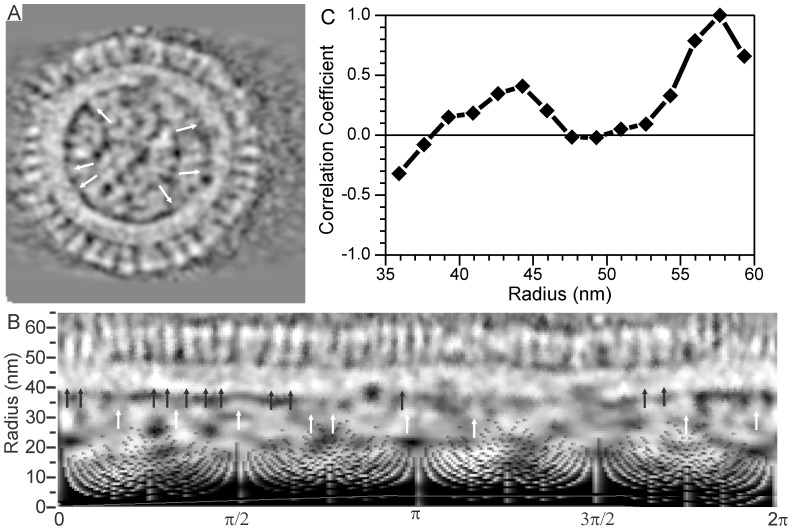
RNP, HA, and NA connections into envelope. A) Central tomogram slice; B) Polar plot of central slice shown in panel A, black arrows show glycoprotein extensions into the matrix and white arrows show contact points between RNPs and matrix. 0° in panel B corresponds to –x direction in panel A and angle increases in counter-clockwise direction. Images are inverted, i.e. higher density regions are lighter. C) Correlation of densities of the outer envelope spikes with their projections into the matrix.

Fig. 7A shows a central tomogram slice of a typical spherical virion. Fig. 7B shows the polar plot (mapped to Cartesian coordinates) of the slice in 7A. Although a polar plot introduces artifacts at small radii (r <25 nm), the Cartesian map facilitates comparisons of density variation in the matrix between greater radial regions (HA and NA spikes) and mid-range radial regions (RNP – matrix contact points). The matrix is the high density band located from 40 nm to 48 nm from the center and is ∼8 nm thick. In Fig. 7B, one can observe higher density regions in the outer section of matrix immediately below the glycoprotein spikes (r = 45 to 48 nm) indicating that the spikes protrude into the matrix. Some of the higher density regions indicative of the surface spike extensions are indicated by black arrows in the Fig. 7B. Below the inner surface of the matrix is a low density band with several regions of higher density, indicated by white arrows in Figs. 7A and 7B. These higher density regions inside the inner region of the matrix are likely locations at which the RNPs are connected to the matrix. The correlation of densities located at r = 57.6 nm (near the surface spike heads) to densities at other radial distances is plotted in Fig. 7C. As expected, a high correlation exists at radii close to the surface spikes heads (55 nm≤r≤60 nm). The correlation is weak (<0.2) at radii corresponding to the membrane (r∼48 nm) due partially to the lower-density lipids. The correlation increases at radial distances corresponding to the matrix (40 nm≤r≤46 nm) confirming the observed extensions of protein spikes into the matrix.

## Conclusions

In summary, the overall morphologies of the B virus particles have been classified by EM. It is demonstrated that a combination of two cryo-EM techniques: (1) analysis of projection images and (2) tomographic reconstruction allows visualization of a B\Lee\40 virus with pleomorphic geometry. The virion structural elements are resolved and their interrelationships revealed. The matrix interacts with both the surface glycoproteins and the RNPs; and may be responsible for organizing the entire viral structure. The HA trimer and NA tetramer were differentiated in the tomograms using ART with blobs reconstructions. The identification and estimation of the number of influenza surface spikes is a valuable parameter in selection of useful B viral strains for successful manufacture of vaccines. Our results demonstrate that the use of blobs shows promise for the high resolution and differentiation of the two surface spikes. This is the first time that tomogram 3D analysis with blobs has been employed to resolve the influenza glycoprotein surface features in context.
